# A Systematic Literature Review of Community-Based Participatory Health Research with Sexual and Gender Minority Communities

**DOI:** 10.1089/heq.2022.0039

**Published:** 2022-08-29

**Authors:** JaNelle M. Ricks, Elizabeth K. Arthur, Shanna D. Stryker, R. Andrew Yockey, Avery M. Anderson, Donald Allensworth-Davies

**Affiliations:** ^1^ Health Behavior and Health Promotion, The Ohio State University College of Public Health, Columbus, Ohio, USA.; ^2^ Equitas Health Institute Midwest SGM Health Research Consortium, Columbus, Ohio, USA.; ^3^ The Ohio State University Comprehensive Cancer, Arthur G. James Cancer Hospital and Richard J. Solove Research Institute, Columbus, Ohio, USA.; ^4^ The Ohio State University College of Nursing, Columbus, Ohio, USA.; ^5^ Family and Community Medicine, University of Cincinnati College of Medicine, Cincinnati, Ohio, USA.; ^6^ Biostatistics and Epidemiology, University of North Texas Health Science Center at Fort Worth, Fort Worth, Texas, USA.; ^7^ Cleveland State University College of Sciences and Health Professions, Cleveland, Ohio, USA.

**Keywords:** community-based participatory research, sexual and gender minority, health disparities, quality measurement

## Abstract

**Purpose:**

The objective was to review sexual and gender minority (SGM) health research studies to gain an understanding of how the community-based participatory research (CBPR) framework has been operationalized.

**Methods:**

We used the Preferred Reporting Items for Systematic Reviews and Meta-Analyses guidelines to conduct a review of all SGM health research studies published in the past 10 years that cited a CBPR approach (PROSPERO Registration No. CRD42016036608). CINAHL, PubMed, and PsycINFO databases were systematically searched in October 2020. Dimensions of community involvement (e.g., shared decision-making; flexibility to community needs and priorities) and the strength of evidence for each dimension were rated using guidance from the Agency of Healthcare Research and Quality.

**Results:**

The 48 eligible articles identified reported a range of 0–11 (out of 13) community elements. Seven studies reported zero elements. Qualitative studies (*n*=28; 58.3%) had an average quality score of 2.32 (range: 1.43–2.5). The 15 (31.3%) cross-sectional studies had an average quality score of 2.08 (range: 1.64–2.27).

**Conclusion:**

Adhering to the CBPR framework is challenging. The benefits of striving toward its principles, however, can move us toward transformative and sustainable social change within SGM communities.

## Introduction

Community-based participatory research (CBPR) is a framework built upon equitable collaboration among scientific researchers, community members, and other stakeholders to improve community health, reduce health disparities, and improve health equity.^1,2^ This adaptive approach engages the community, recognizes and leverages the diverse strengths and contributions of all research partners, and is action oriented in that it seeks not only to understand problems but also to propose cocreated solutions. CBPR principles include the following: colearning between academic and community partners; capacity building and empowerment; mutually beneficial knowledge and findings; bidirectional leadership and decision-making; and long-term commitment.^3,4^

CBPR shifts the traditional research paradigm which focuses on a specific set of research methods or techniques, largely developed in academic settings, by prioritizing the relationship between academic and community-based research partners and the creation of positive, transformative, and sustainable social change within communities. This represents a systematic effort to incorporate community participation, decision-making, and practices into the research practice.^5^ Actively integrating community members during all phases of the research project helps to ensure that the methods used and data collected are culturally grounded and reflect the lived experiences of the population.^6,7^ Community integration within research teams also has the potential to ensure that health research is acceptable and directly relevant to target communities, potentially improving the rigor of these research efforts overall.

CBPR has been used to examine a variety of health topics such as mental health, food insecurity, diabetes, homelessness, and HIV. It has also been used to assess community characteristics which are key to partnership sustainability, such as capacity, readiness, social capital, and empowerment.^8^ It can be adapted for diverse community collaborations and may be particularly valuable for work with vulnerable and historically underserved communities.^9–11^

Sexual and gender minority (SGM) is an inclusive term used to refer to a diverse array of people who are gay, lesbian, bisexual, transgender, queer (LGBTQ), gender non-binary or non-conforming, two-spirit, asexual, pansexual, intersex, and other sexual orientation and gender identities (SOGIs). National surveys estimate that ∼4–7% of the United States population (or 11 million people, roughly equivalent to the population of Ohio) are SGM but are likely underestimates given the failure of survey efforts such as the U.S. Census or American Community Survey to robustly collect this information.^12,13^

Due to discrimination and social marginalization, SGM people are at risk for poor health behaviors and health outcomes.^14,15^ Compared to their cisgender and heterosexual counterparts, SGM people experience higher rates of HIV infection and other sexually transmitted infections, smoking, drug and alcohol use, and mental health problems.^14^ There is evidence that sexual minority women have higher odds of risk factors for hypertension, diabetes, and breast cancer.^14^ Transgender adults may have higher risk factors for cardiovascular disease and myocardial infarction.^14^ However, due to lack of data collection regarding sexual orientation and gender, the full extent of health disparities experienced by SGM people is not known.^14^

Health disparities in SGM people are not caused by their gender or sexuality, but by the discrimination, minority stress, and sociopolitical barriers to optimal health that lead to exposures and behaviors known to contribute to disease and disability.^16,17^ For example, SGM people face barriers to accessing basic health care across the life span, and many experience discrimination or refusal of service when seeking health care.^18^ SGM people are also more likely than cisgender, heterosexual people to face employment discrimination and lack health insurance.^18^ Even when care is accessed, health care providers are often underprepared to provide affirming health care to SGM people.^19,20^

SGM communities are heterogeneous, and intersectionality must be considered. SGM individuals differ markedly by not only SOGIs but also in life experiences by age cohort, racial/ethnic group, socioeconomic strata, disability/ability, and immigration status. Integrating intersectionality into health disparity research emphasizes the need to consider this diversity in research, health care, and policy given its influence on an individual's health risks, screening behaviors, and treatment experiences. Intersectionality, a feminist sociological theory, considers the intersection of marginalized or minoritized identities^21^ and how multiple oppressions coexist and interact on various and often simultaneous levels. Intersectional disparities among SGM have also been documented by older age,^22^ lower socioeconomic status,^23^ and immigrant status,^24^ among others.

SGM communities have historical contexts for community-engaged research. ACT UP (AIDS Coalition to Unleash Power) was formed in response to social neglect, government negligence, and complacency of the medical establishment during the 1980s.^25^ ACT UP has advocated for sustained investment in HIV/acquired immune deficiency syndrome (AIDS) treatment and related coinfection research, equitable access to HIV/AIDS prevention and care, and tackling structural drivers (e.g., stigma, discrimination, and poverty) of the HIV/AIDS epidemic.

To make meaningful, impactful progress in SGM health equity, researchers must understand the influence of social determinants on SGM health, as well as the priorities and behaviors of this community, from their perspective. CBPR is well-positioned to more equitably include SGM communities in the pursuit of transformational research outcomes.^1,2,26^ CBPR also has the potential to address noted gaps in SGM health research topic diversification. Although efforts have been made to establish a more comprehensive national SGM research agenda, HIV remains disproportionately prioritized, leaving gaps in other areas such as chronic disease and comorbidity, aging, methods and measurement, and social determinants of health (SDOH).^27,28^

It has been beneficial in the development of tailored assessment tools for specific populations^29,30^; assessment of a wide range of chronic health conditions^31–33^; and addressing the social determinants impacting the health of minoritized communities.^34–37^

Although the incorporation of CBPR methodology into SGM health research likely has profound benefits, CBPR practitioners face a number of challenges which threaten to limit full implementation of CBPR principles. For example, uncurtailed community-academic partner power differentials, conflicting visions about the work, and limited structural support from funders and academic institutions may lead to minimal community engagement.^2,38,39^ To effectively utilize CBPR to produce findings, knowledge, and outcomes with maximum benefit to SGM people, it is particularly important to characterize community involvement in the research process.

This systematic literature review contributes to the knowledge base by identifying gaps in implementation that limit SGM community members from becoming full partners, contributing their unique knowledge and experiences while also shielding their interests. We present the results of a review of peer-reviewed SGM health studies published over the past 10 years, which describe a CBPR or community-engaged approach, to gain a clear understanding of how CBPR has been operationalized in SGM communities across the United States.

## Materials and Methods

This systematic literature review was conducted in accordance with the Preferred Reporting Items for Systematic Reviews and Meta-Analyses^40^ and has been registered with PROSPERO (Registration No. CRD42016036608), an international database of prospectively registered systematic reviews.^41^ Articles were included in the review if they described original SGM health research studies conducted in the United States with mention of CBPR or community-engaged methods and were published between January 2010 and October 2020. “Health research” was categorized broadly, allowing for “health adjacent” topics such as SDOH. Editorials, systematic review articles, meta-analyses, case studies, and methodological articles were excluded.

We searched article titles and abstracts in the CINAHL, PubMed, and PsycINFO databases using keyword combinations specific to CBPR and SGM health research (Supplementary Appendix SA1). The date of the last search was October 12, 2020. Article screening, extraction, and assessment of CBPR criteria were conducted using Covidence^®^, a web-based software for management of systematic literature reviews.^42^

Each of the articles included in the final review was independently assessed by two members of our research team with the reviewers meeting to discuss and reach consensus on discrepant items. We also met as a research team to ensure that data extraction elements and quality criteria were understood and applied consistently during the review process. Data extraction and assessment of CBPR criteria were conducted using the data elements recommended by Viswanathan et al. in their report on assessing CBPR evidence for the Agency of Healthcare Research and Quality (AHRQ) (Supplementary Appendix SA2).^43^ Assessment of SGM community involvement comprised eight CBPR elements: (1) shared decision-making; (2) community participation barrier removed; (3) socioeconomic determinants of health addressed; (4) flexibility to community needs and priorities; (5) capacity building; (6) findings disseminated to participants; (7) findings applied to health-related intervention or policy change; and (8) intervention sustainability.^43^

A quality assessment was also conducted to classify each article as good, fair, or poor quality (Supplementary Appendix SA3).^43^ Quality of each element was rated on a scale of 1–3. One indicated that the article provided insufficient information or that element was poorly captured. A score of three represented the highest quality. A final score (range: 1–3) was calculated by averaging the scores of all relevant quality elements. Scores were assigned based on what was described in the article and, thus, may under represent elements included but not described in the study design.

The general SGM community was not engaged in the design or conduct of this systematic review. Insofar as the intended audience was SGM health researchers, our author panel comprised members of the Equitas Health Institute Midwest Health Research Consortium, who are all SGM health researchers.

### Reflexivity statement

The authors of this article include gender diverse (cisgender, transgender, and/or non-binary) and sexuality diverse (queer, gay, and straight) researchers who are nurses, a physician, and public health experts in SGM health.

## Results

### Overview

**FIG. 1.**
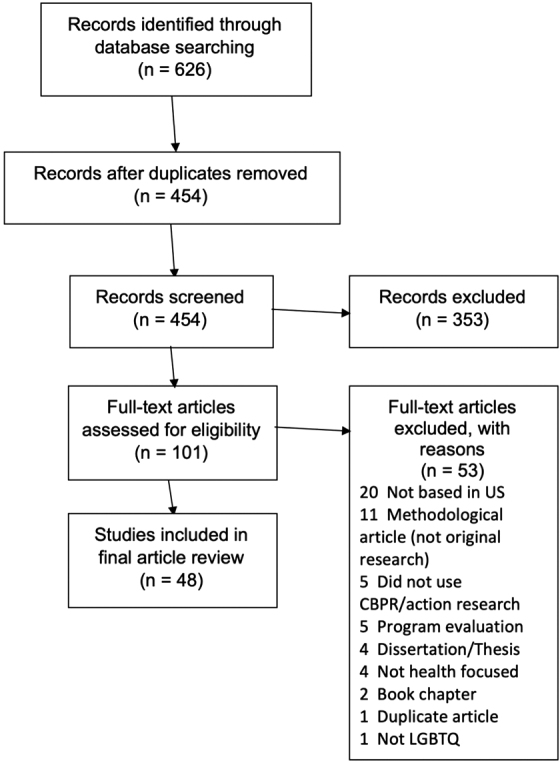
PRISMA flow diagram of sexual and gender minority health CBPR studies review. CBPR, community-based participatory research.

Forty-eight studies^44–91^ were identified for inclusion in this review, as outlined in Figure 1. A search of the databases revealed 454 nonduplicated records, 353 of which were irrelevant based on a review of the titles and abstracts. Of the remaining 101 relevant records, 20 described studies done outside of the United States, 11 were methodological articles which did not share the results of original research, 5 did not use CBPR/action research approaches, 5 described program evaluation only, 4 were dissertations, 4 were related to topics other than health (e.g., education), 2 were book chapters, 1 was a duplicate article not identified on initial screening, and 1 was unrelated to health. As seen in Table 1, there has been a steady increase in the number of articles published on this topic.

**Table 1.** Characteristics of Included Sexual and Gender Minority Health Community-Based Participatory Research Studies (*n*=48)

Characteristic	Number of studies (%)
Year of publication	
2010–2011	7 (14.6)
2012–2013	4 (8.3)
2014–2015	10 (20.8)
2016–2017	7 (14.6)
2018–2019	11 (22.9)
2020^a^	9 (18.8)
Primary topic area	
HIV/AIDS	14 (29.2)
Transgender health	13 (27.1)
Health care access	8 (19.7)
Mental health	7 (14.6)
Youth services	6 (12.5)
Sexual health	5 (10.4)
Substance use	5 (10.4)
Older adult services	4 (8.3)
Physical health	4 (8.3)
Number of funding sources	
None listed	14 (29.2)
1	20 (41.7)
2	8 (16.7)
3 or more	6 (12.5)
Types of funding sources	
Federal	25 (52.1)
State	3 (6.3)
University	20 (41.7)
Private foundations or sources	8 (16.7)

^a^ Literature search conducted in October 2020.

AIDS, acquired immune deficiency syndrome.

### Study design

The included studies are described in Table 2. Most of the studies were qualitative (28; 58.3%) or cross-sectional (15; 31.3%). Two reported the results of randomized controlled trials (RCTs), and another described a secondary review of survey data obtained during one of the aforementioned RCTs. All three of the articles sharing results from RCTs were done by the same CBPR partnership in North Carolina.

**Table 2.** Summary of Included Sexual and Gender Minority Health Community-Based Participatory Research Studies by Number of Community Involvement Elements (*n*=48)

Abbreviated citation	Study design	Primary topic area	Sample					Study setting	No. of community involvement elements (0–13)	Average quality score (0–3)
			Sample description	Inclusion		Age, range	Total, ***N***			
				Bisexual	TGN					
Perone et al.^66^	Qualitative	Older adult services	Nine participants (45%) were lesbian, 5 (25%) gay, 4 (20%) bisexual, and 1 (5%) queer. Eight participants (38.1%) were people of color, 5 (23.8%) specifically African American or Black.	Y^a^	Y^a^	19–78	21	A large Mid-western city, including four rural and urban counties	11	1.69
Rhodes et al.^72^	Cross-sectional	HIV/AIDS	71.2% were gay, 3% heterosexual or straight, and 25.8% bisexual. 56.4% were White, 37.6% Black or African American, 1% were Alaskan native/American Indian, 0.5% Asian. 8.6% reported being HIV-positive, and 14.8% reported never having been tested for HIV.	Y	N	18–61	210	Northwest North Carolina	10	1.93
Smith et al.^81^	Qualitative	Mental health Transgender health	Transgender adults; male (30%), female (50%), non-binary (20%), two spirit (6.7%); 76.6% Caucasian, Latinx/Hispanic (6.6%), Native American (13.3%), Pacific Islander (3.3%)	NR	Y	18–67	30	Rural Montana	9	2.05
Stewart et al.^83^	Qualitative	Transgender health	Participants were attendees of trans/non-binary health summits or recruited online. Gender diverse participants (*n*=96) were men and/or trans men (31%); women and/or trans women (36%); or a gender included under non-binary (32%). Forty-six percent were bisexual/pansexual/queer and 20% heterosexual. Twenty percent were non-White and 8% were of Hispanic, Latinx, or Spanish origin. Cisgender ally attendees (*n*=29) were heterosexual (48%), gay/lesbian (17%), and bisexual/pansexual/queer (28%).	Y	Y	13+	125	Arkansas	9	2.26
Rhodes et al.^69^	Qualitative	Transgender health	Immigrant Latina transgender women. All were originally from Mexico. Mean number of years living in the United States was 10.	NR	Y	22–45	9	North Carolina	8	2.48
Boggs et al.^47^	Qualitative	Older adult services	Participants from at least one data collection event: intercept surveys (*n*=17); focus groups (*n*=14); town hall meeting (*n*=30); or final interview (*n*=12). Mostly gay males in their 70s and lesbian females in their 60s.	Y^a^	Y^a^	40–79	73	Denver, CO	7	1.95
Bryant et al.^48^	Qualitative	Substance use	The focus groups included a total of 36 participants, who were 42% African American, 58% White, 58% gay male, 36% lesbian, and 6% transgender (male-to-female). Groups organized by smoking status: current smokers, former smokers, and nonsmokers. Thirty participants involved in community meeting.	NR	Y^a^	23–58	66	Atlanta, GA	7	2.14
Felner et al.^51^	Qualitative	Youth services Health care access	11 youth researchers, most were people of color and all were LGBTQ; 26 current and former LGBTQ service patrons; and 10 LGBTQ service providers at 1 of 6 services.	Y	Y^a^	18–24	48	Chicago, IL	7	2.41
Felner et al.^52^	Qualitative	Youth services	LGBTQ young adults (*N*=26) who utilize support network in Chicago and service providers (*N*=10) at LGBTQ-supportive youth programs. Focus group participants were mostly Black and/or African American (80%) and male (58%).	Y	Y^a^	Focus group: 20–29	36	Chicago, IL	7	2.45
Rhodes et al.^70^	Randomized controlled trial	HIV/AIDS	Immigrant Spanish-speaking Latinx GBMSM and TW social network members. Most participants were foreign born. 66.3% speaking only/mostly Spanish. Foreign-born participants had been living in the United States for a mean of 10.1 years. Seventy-three percent were undocumented. Eighty percent gay, 16% bisexual, 11% transgender.	Y	Y	18–48	166	North Carolina	7	2.39
Schnarrs et al.^79^	Cross-sectional	Sexual health	Rural MSM; 55% gay, 20% bisexual, 17% heterosexual. Participants were White (89.6%), African American/Black, Asian/Pacific Islander (1.3%) or of another race (2.6%), and Hispanic/Latino (2.3%). The majority (81.2%) resided in the largest city (population of 69,291 persons), with the remaining from surrounding communities of that area (12.0%).	Y	N	18–67	309	Rural Indiana	7	1.86
Hergenrather et al.^55^	Non-randomized experimental	HIV/AIDS	African American men, who were gay and unemployed, participated.	N	N	37–57	7	Washington, DC	6	2.26
Pelster et al.^65^	Cross-sectional	Substance use	Participants were 59% male, 75% homosexual, 90.2% White, 93.8% non-Hispanic. A small percentage were either Alaskan native/American Indian (4.8%) or Asian (0.7%).	Y	Y	19–70	763	Nebraska	6	2.30
Rhodes et al.^76^	Quasi-experimental	HIV/AIDS	Participant of an online chat room for MSM; 58% were gay; 18% were bisexual; 24% did not report sexual identity. The majority were White/European (71%); and 1.6% reported HIV positivity.	Y	N	18–78	346	Northwest North Carolina	6	2.26
Rodríguez-Díaz et al.^77^	Cross-sectional	Health care access	LGBT Pride Parade participants. 59.7% were male and 39.9% female. One person was transgender. 56.0% identified as gay, 34.5% lesbian, 7.8% bisexual, and 1.7% other.	Y	Y^a^	18–63	233	San Juan, Puerto Rico	6	1.86
Solorio et al.^82^	Qualitative	HIV/AIDS	Latino immigrant MSM, more than 75% Mexican descent; Spanish-speaking (monolingual). Most resided in the United States for <5 years.	N	N	18–40	66	Seattle, Washington	6	2.26
Martinez et al.^59^	Qualitative	Sexual health	25 non-Latino White, 25 non-Latino Black, and 25 Latino men. All men identified as “behaviorally bisexual.” Nearly all men (96%) were born outside the United States. In addition, most men (72%) originally migrated to the United States from urban areas, and most (72%) had been living in the United States for <10 years.	Y	N	19–70	25	Indianapolis and the surrounding catchment area	5	2.00
Rhodes et al.^71^	Qualitative	HIV/AIDS	Immigrant Latino MSM. Over 85% reported Mexico as their country of origin. Majority was “gay” or “homosexual,” two were “bisexual,” and one was ‘heterosexual’. Four participants reported sex with both women and men during the past 3 months. Three participants were HIV positive; and two were male-to-female transgender.	Y^a^	Y^a^	18–48	21	Rural North Carolina	5	2.36
Rhodes et al.^73^	Qualitative	Sexual health	Nine focus groups include MSM participants (*n*=88) who were African American/Black (*n*=28), Hispanic/Latino (*n*=33), White (*n*=21), and biracial/ethnic (*n*=6). Community forum attendees included community members (*n*=4), service organization leaders (*n*=15), and others; two historically Black colleges (*n*=3); and two academic research institutions (*n*=4). Included males and females, gay and non-gay attendees.	N	N	18–60	122	Northwest and Central North Carolina	5	2.10
Van Wagenen et al.^90^	Qualitative	Older adult services	LGBT adults age 60 and older. Half the sample was female; one was transgender. The vast majority (91%) of the sample was gay or lesbian, one was bisexual, and one was heterosexual. Eighteen percent were African American, remainder NHW.	Y^a^	Y^a^	60–80	22	Boston, MA	5	1.91
Alio et al.^44^	Qualitative with survey	HIV/AIDS	Leaders and Prominent members of the House Ball Community; African American, Latino, Afro-Latino or Afro-Caribbean, MSM or Transgender female.	Y^a^	Y^a^	25.4	14	Rochester/Buffalo region of New York	4	2.27
Alonzo et al.^45^	Randomized, controlled intervention with qualitative data	HIV/AIDS	Hispanic/Latino MSM or Hispanic/Latina transgender women.	Y	Y	18–55	152	North Carolina, USA	4	1.79
Hussen et al.^56^	Qualitative	HIV/AIDS	Young, Black gay or bisexual MSM (*N*=29) and health care or social service providers (*N*=28).	Y	N	18–29	57	Not reported	4	2.14
Mountz^62^	Qualitative	Youth services Transgender health	LGBT youth of color with the experience of having been incarcerated in a “girls” juvenile justice facility.	Y	Y	18–25	10	New York City and Duchess County	4	2.27
Vissman et al.^91^	Qualitative	Health care access	Latino community members and health service providers. Community members: 20 (56%) men, 14 (39%) women, and 2 (6%) male-to-female transgender participants. Seven men were gay. 69.4% were born in Mexico and 13.9% in the United States; others were foreign born outside Mexico. Service providers: one physician and one pharmacist serving mostly rural Latinos living in NC, one AIDS case manager, one domestic violence case manager, one medical interpreter for a local public health department, and one community health educator.	NR	Y^a^	23–64	36	Five rural counties in central NC	4	2.15
Reif et al.^68^	Pilot feasibility study	HIV/AIDS Mental health	Individuals living with HIV and a mental health disorder; 35% female; 80% African American.	N	N	NR	40	Charlotte, North Carolina	3	2.19
Rhodes et al.^75^	Cross-sectional	Sexual health Substance use	Rural immigrant Latino MSM, nearly 80% from Mexico. Sixteen percent transgender; 89% gay, 10% bisexual.	Y	Y	18–48	190	Rural North Carolina	3	2.24
Salkas et al.^78^	Cross-sectional	Transgender health Health care access	Transmasculine (*n*=31) and transfeminine (*n*=46) participants. 7.8% people of color.	NR	Y	NR	77	Online survey recruitment through Wisconsin-based venues/groups	3	1.64
Stover et al.^84^	Qualitative	Health care access	Students from 13 colleges/universities. Cisgender college students were lesbian (*n*=7), gay (*n*=7), and bisexual (*n*=4; all females).	Y^a^	N	19–24	18	New England	3	1.70
Strang et al.^85^	Qualitative	Transgender health Youth services	Autistic/neurodiverse gender-diverse youth (*n*=31) and their parents (*n*=46) connected to services at a large medical center. Parents were 30 mothers and 16 fathers; 15 of these parents, all in heterosexual relationships, participated as couples. Of participating youth, 16 were trans-female, 11 trans-male, and 4 non-binary-transgender (assumed female at birth). Twenty-seven youth were White, two Asian, two mixed-race, and two Hispanic/Latinx. Input from key stakeholders or expert clinical providers was obtained but they were not involved in the intervention.	NR	Y	Youth: 12–19	77	Washington DC	3	2.32
Kattari et al.^58^	Cross-sectional	Transgender health Mental health	TGD adults.	Y	Y	28.6	659	Michigan	2	2.09
Noonan et al.^64^	Qualitative	Transgender health Health care access	University of Louisville School of Medicine faculty, staff, medical students, community health professionals, and community members. Fifty-nine participants in the forum and 100 completed follow-up survey.	NR	Y	NR	159	Louisville	2	2.08
Sun et al.^86^	Cross-sectional	Mental health	Immigrant adult Latino sexual minority men or transgender women. Average time in United States 10.1 years, 80.6% male, others female, male-to-female transgender, or transvesti.	N	Y	18–61	186	North Carolina	2	2.32
Sun et al.^87^	Cross-sectional	Sexual health	Participants were MSM and/or transgender adults. Most were White (82.3%) and male (98.7%). Included gay (45%), bisexual (40.7%), and heterosexual/other (13.6%) participants.	Y	Y	18–74	457	Four metropolitan areas across North Carolina	2	2.14
Tanner et al.^88^	Cross-sectional	Health care access	Immigrant Latino gay and bisexual men, MSM, and transgender people. Included community lay health advisors (*Navegantes*) and participants from the social network of Navegantes. 18.3% of the population identified as transgender.	N	Y	18–61	180	North Carolina	2	2.18
Hardacker et al.^54^	Qualitative	Physical health	Adults were assigned female gender at birth and now identify as gender non-conforming, queer, transgender men, lesbian, or bisexual. A small percentage were either Alaskan native/American Indian (2.8%) or Asian (8.3%).	Y	Y	18–64	36	Not reported	1	2.09
Irwin et al.^57^	Cross-sectional	Mental health	LGBT adults.	NR	Y	19–70	770	Nebraska and Iowa	1	2.27
Proctor and Krusen^67^	Qualitative	Older adult services	Older LGBTQ veterans.	Y^a^	Y^a^	51–87	7	Pacific Northwest	1	1.43
Rhodes et al.^74^	Qualitative	HIV/AIDS	Randomly selected GBMSM and TW with HIV who had completed an intervention to improve HIV care engagement participated in the interviews. Six participants were Black/African American, five were Spanish-speaking Latinx, and four were White. Gay (14) and bisexual (1).	Y^a^	N	Mean age 28	15	Guilford County, NC	1	2.10
Schnarrs et al.^80^	Cross-sectional	Mental health Physical health	27.2% TGD; gay (37.8%), lesbian (25.8%), bisexual/pansexual (26%), other sexuality (10.4%); White (36.7%), Latinx/Hispanic (45.3%), Black/African American (7.1%); Other race (10.9%)	Y	Y	18+	477	San Antonio, TX	1	2.05
Teti et al.^89^	Qualitative	Mental health Physical health Transgender health	Participants were transmasculine young adults who had not undergone surgical procedures. Fourteen were White, one Hispanic, and one Black.	NR	Y	19–25	16	Small Midwestern city	1	1.50
Austin and Craig^46^	Qualitative	Youth services	Culturally diverse SGMY at three high schools were Hispanic (*n*=14), Haitian (*n*=7), African American (*n*=4), and other Caribbean (*n*=3). Participants were female (*n*=23), male (*n*=4), and other (*n*=1). Sexual orientations were bisexual (*n*=12), lesbian (*n*=7), mostly heterosexual (*n*=5), gay (*n*=3), and pansexual (*n*=1). Informed stakeholders (*n*=6), including clinicians, service providers working with SGMY in South Florida.	Y	NR	<18	34	Miami, Florida	0	1.94
Edelman et al.^49^	Qualitative	HIV/AIDS	Local community medical case managers (*n*=14), disease intervention specialists (*n*=7), and MSM (*n*=24).	Y	N	42.5	45	Connecticut	0	2.50
Edelman et al.^50^	Qualitative	HIV/AIDS Substance use	Medical case managers (*n*=14), disease intervention specialists (*n*=7), and MSM (*n*=17).	Y	N	46	38	Connecticut	0	2.44
Fisher et al.^53^	Cross-sectional	Substance use Physical health	Participants (*N*=723) were from Nebraska and Council Bluffs, Iowa. The majority of participants resided in an urban area (89.5%) and were White (91.6%). Rural participants (*n*=75) were predominantly male (*n*=49, 65.3%) and White (*n*=71, 95.9%). A small percentage were either Alaskan native/American Indian (0.8% or Asian 0.9%). More rural participants were bisexual (21.3% vs. 15.9%), while rates of transgender identity were similar between rural and urban populations (10.7% vs. 10.8%). All persons who were heterosexual also were transgender.	Y	Y	19+	723	Nebraska and Iowa	0	2.14
Martinez-Velez et al.^60^	Cross-sectional	Transgender health Health care access	Transgender and gender non-conforming individuals.	Y^a^	Y	15–49	52	Puerto Rico	0	2.05
Meyer et al.^61^	Qualitative	Transgender health Health care access	TGD adults.	NR	Y	22–64	27	Nebraska	0	2.09
Mountz et al.^63^	Qualitative	Youth services Transgender health	TGD youth with experience in the foster care system. All were racial/ethnic minorities.	Y	Y	18–25	7	Los Angeles, California	0	1.64

^a^ Y^*^=yes *n*≦5.

GBMSM, gay, bisexual and other men who have sex with men; LGBTQ, lesbian, bisexual, transgender, queer; MSM, men who have sex with men; N, not included; NR, not reported; SGMY, sexual or gender minority youth; TGD, transgender and gender diverse; TW, transgender women; Y, yes.

### Study quality

Among the 28 qualitative studies included in this review, the average quality rating ranged from 1.43 to 2.5, with an average score of 2.32. Among the 15 cross-sectional studies included in this review, the average quality rating ranged from 1.64 to 2.27, with an average score of 2.08.

### Study characteristics

#### Participants

Study sample size ranged from 7 to 763 participants (Table 2). Three studies (6.3%) exclusively included older adults, although the lower age limit for “older adult” ranged from 40 to 60 years old.^47,67,90^ Sixteen studies (33.3%) exclusively included the perspectives of people of color (POC); one of these recruited only Black men who have sex with men living with HIV,^55^ and nine focused on the health of Latinx SGM individuals. In an additional eight studies (14.6%), White non-Hispanic participants were the minority.

There was a wide variety of SGM identities represented within the 48 included studies. As seen in Table 2, 32 studies (66.7%) included bisexual participants, although 9 of these had 5 or less bisexual participants. Ten studies (20.8%) included lesbian participants, but no study focused solely on experiences of these women. More than half of studies in this review (*n*=33, 68.8%) included transgender/gender non-conforming (TGNC) individuals. Nine of these included exclusively TGNC participants and 10 included 5 or less TGNC participants.

#### Primary topic

Reviewed studies covered nine primary topics (Table 1). The most commonly addressed were HIV/AIDS (12/48, 25%) and transgender health (12/48, 25%). Access to health care was also commonly explored (8/48, 16.8%). Four studies (8.3%) specifically address older SGM health services, and six studies (12.5%) addressed SGM youth services. Other health topics examined included: physical health (four studies; 8.3%), sexual health (five studies; 10.4%), mental health (seven studies; 14.6%), and substance use (six studies; 12.5%). Sixteen of the studies addressed two primary topics (33.3%).

#### Funding source

A sizable proportion of studies (29.2%) reported no funding source (Table 1). Five (10.4%) had three or more funding sources, eight studies (16.7%) had two funding sources, and one study (2.1%) had one funding source. Of the funded studies, 25 (52.1%) received Federal funding, 3 received State funding (6.3%), 20 were funded through university mechanisms (41.7%), and 8 (16.7%) received funding from private foundations or other sources.

### Elements of community involvement reported

Within the reviewed CBPR studies, a wide range of community involvement was reported. The distinction between academic and community partners was not always precise because some descriptions of community partners included academic partners with SGM identities. The number of community elements incorporated ranged from 0 to 11, with seven studies not specifically describing any of the AHRQ-determined community involvement elements in their article. The study with the most community elements was a qualitative study examining health services among older adults.^66^ Examples of elements were: selecting the research question; developing the research proposal; collecting the data and other implementation activities; interpreting, disseminating, and applying the findings.

The most commonly reported community involvement elements were recruitment (56.3%) and study design (52.1%) (Table 3). Furthermore, more than half (54.2%) reported community partners assisting with interpretation of the findings. The research question of 21 projects (43.8%) was selected in partnership with community members, but only 3 (6.3%) study proposals were developed with community partner input. A small majority (4.2%) described community partners assisting with retaining the participants. No study reported shared financial responsibility with community members.

**Table 3.** Community Involvement in Sexual and Gender Minority Health Community-Based Participatory Research Studies (*n*=48)

Element of community involvement	Number of studies (%)
Recruit subjects	27 (56.3)
Interpret findings	26 (54.2)
Design study	25 (52.1)
Develop surveys/instruments	24 (50)
Select research question	21 (43.8)
Data collection	19 (39.6)
Disseminate findings	14 (29.2)
Apply findings	13 (27.1)
Develop interventions	11 (22.9)
Implement interventions	5 (10.4)
Develop proposal	3 (6.3)
Retain subjects	2 (4.2)
Have financial responsibility	0 (0)

## Discussion

Despite wider recognition of the value of community-academic partnerships in SGM health research over the past decade, this work remains sparse and adherence to the CBPR framework is variable given inherent challenges and an understanding that a single set of principles may not be appropriate for all communities and all contexts.^4^ On the continuum of community-engaged research, CBPR moves beyond community involvement and collaboration and signifies the highest level of engagement. It is hallmarked by shared leadership and a strong and sustained partnership that ideally integrates the community into each phase of the project.^92^ Of the 48 studies identified in this review, none met this standard. No study included all 13 community involvement elements identified in the 2004 AHRQ report, and 7 studies included none at all.^46,49,50,53,60–62^

Of note, only those elements described in the articles were assessed, and it may be likely that limitations such as article word limits may account for incomplete descriptions of methods and community involvement. Among the four studies with the greatest number of elements, the quality varied, with no study achieving an optimal assessment score.^60,69,81,83^

Considering all studies, community involvement was disproportionately concentrated in the study design, recruitment, instrument development, and interpretation of results. In most cases, the community did not select the research topic and/or the research question, an activity essential to creating a shared vision and ensuring the project is community driven. Excluding the community at this critical point can limit identification of priorities and outcomes most important to them and instead promote the researcher's agenda and expertise or the funder's priorities.^2^ Diminishing bidirectional collaboration and power sharing also damages the community–researcher relationship and can create a climate that the community does not perceive as open and just. This impacts not only the active study but also may propagate sentiments of distrust that negatively impact future research efforts.

There was limited evidence that studies prioritized removing barriers to community participation. The most commonly reported strategies included the provision of monetary incentives and transportation assistance (i.e., bus pass). Although these may address some challenges, other social and structural barriers may also impede participation of community members in the research process. Academic partners have the opportunity to build community capacity by providing education and resources for navigating research processes (Table 4).

**Table 4.** Recommendations for the Advancement of Sexual and Gender Minority Community-Based Participatory Research

For researchers
Prioritize removing barriers to community participation
*Hold meetings in community-accessible locations at amenable times* *Provide education and resources for navigating the research process*
Involve the community in selection of research topic/question
*With special consideration of less explored areas: demographic research, intervention research, social influences, and health inequities*
Pay thoughtful attention to intersectional effects of marginalized identities
*Use demographic measurement tools that account for the vast diversity within the LGBTQ+ community*
Supplement, but do not replace, a community member of lived experience with relevant community partner(s) and stakeholder(s)
Ensure capacity building, partnership sustainability, and use of findings for policy change
For academic institutions
Allow for promotion/tenure timetable alternatives with CBPR engagement
Invest in fostering sustainable community-academic partnerships
Require curriculum training in community-based research methodology
For funders
Move beyond the rigid model of preprogram budgets
Offer flexible funding opportunities to support extended time needed in CBPR
Enable greater flexibility in the choice of topics
Increase availability of funding models with multiple streams, alternative deliverables, and structures with flexibility to adapt to emerging community needs

CBPR, community-based participatory research.

Defining “community” was critical to understanding how authentically each study's findings may have represented the SGM experience. We therefore limited our definition of community to include SGM individuals representing the community to be served by the research efforts. In several studies reviewed, researchers and/or community advisory board members included stakeholder groups such as SGM service organizations and clinical providers treating SGM patients; this alone was not sufficient to warrant community involvement.

Difficulty in recruiting a sufficient number of SGM community members is not unusual in health research, particularly when working in a small geographically defined community or with some subpopulations (e.g., TGNC). In this case, supplementing the community member group with relevant community leaders and stakeholders can be helpful. Similarly, some studies focused on exploration of topics for which the health care and/or service provider perspective was relevant. But because it could not be assumed that all stakeholders who participated were well qualified to speak with authority about the SGM experience, if no SGM community members with lived experience with the health topic of interest were involved in a particular study element, credit was not given for that element.

SGM POC, bisexual, and transgender people remain inadequately represented on the general SGM health landscape. Within CBPR studies, however, a greater proportion of these populations were represented. Twenty-five studies reported mostly or entirely POC samples, with significant representation of Latinx and Black/African American participants. Over half of studies included bisexual participants, although only one focused exclusively on bisexual health despite evidence that most sexual minority adults, particularly younger cohorts, identify as bisexual.^93^ Twenty-five percent examined transgender mental health and health care use and access experiences. Few studies, however, included gender diverse participants such as genderqueer, gender non-binary, and gender non-conforming individuals.^54,60,66,83^ Intersectional perspectives of some groups such as immigrant Latinas, juvenile justice-involved girls, and autistic and neurodiverse youth were also included.

Despite these strengths in representation, inconsistencies in SOGI conceptualization, measurement, and operationalization were observed across studies. Variations were likely influenced by study timing (i.e., language reflective of conventions of the time), geographic region, and knowledge and preferences of research teams (it is unclear if SGM partner input was integrated as no studies explicitly reported this). For example, some studies recruited under the broad “LGBTQ” umbrella without disaggregating sexuality and gender subgroups.^51,52,62,67,80^ Sex and gender are often mistakenly conflated with the assumption that they do not differ from each other.

Of note, only two studies explicitly reported representation of indigenous third gender/non-binary roles (i.e., two-spirit, fa'afafine, māhū).^62,81^ Our operationalization of SOGI was not entirely inclusive, and the search structure used missed relevant publications as we failed to include the appropriate MeSH terms and keywords—a major limitation. In addition, it is likely that people who endorsed these identities comprised a small proportion of total participants in included studies and were reported under “other” and “additional” SOGI categories.

Aggregating the diversity under the LGBTQ+ umbrella risks mischaracterizing experiences of power, sexuality, and relations. SOGIs are core aspects that shape opportunities and experiences of discrimination that influence health; therefore, accurate conceptualization and measurement is crucial. Continued efforts have been made to improve measurement of sex, gender, and sexual orientation, most recently, the groundbreaking 2022 National Academies of Science, Engineering, and Medicine report.^94^ This report is the most comprehensive to date of measurement-related research for these constructs. As utilization of these recommendations permeate the research community, data collection can be enhanced, demonstrating respect to and making visible the SGM participant, partner, or patient. Dissemination will also enable data harmonization between data sources, further building the bodies of work that will inform future health interventions and practice.

SGM CBPR health research covers a range of topics. Although our definition of “health” research was expansive enough to include topics such as mental health and SDOH, it is possible that other relevant health-related topics were excluded from consideration, limiting the results. In addition, we did not search gray literature, which might have resulted in the identification of additional studies that would have been eligible for inclusion. In studies identified there was a concentration in HIV/AIDS, reflective of patterns in SGM health research overall. More research is needed in a large number of diverse areas, including demographic research, intervention research, social influences, and health inequities.^28^ Use of CBPR can play a key role in addressing these gaps, particularly exploration of approaches to addressing the SDOH.^2^

The majority of studies reviewed were exploratory or descriptive with either a qualitative or cross-sectional survey design. This decreased overall community involvement scores as credit could not be given for activities such as intervention implementation or participant retention. In addition, capacity building, partnership sustainability, and use of findings for policy change were commonly lacking, independent of study design. These are significant and interrelated gaps. Moving the needle from exploratory to interventional research can provide a foundation of growth, sustainability, and innovation within a community that over time can enable transformational change to occur. To facilitate this shift, commitment from funders – particularly those of federal mechanisms—is integral.

CBPR studies are often unfunded, as was the case for 30% of studies in this review, or funded by smaller mechanisms, offering little-to-no incentive or sustainable support to engage in robust CBPR work. Adequate and flexible funding opportunities are needed to support the extended time needed upfront to build relationships with community partners, as well as the additional time—months or years—often involved as the community participation process evolves. Increased success has been demonstrated in programs that have had access to “braided funding” from multiple streams, a model that affords more flexibility in terms of concrete “deliverables” and provides programs the authority to fund efforts that are based on emerging community needs.^95^ Similar flexibility should be explored and expanded in other government and foundation research grant programs to move beyond the rigid model of preprogram budgets.

Other structural and educational barriers to CBPR work have been documented (Table 4) and may have limited CBPR projects included in our review. For example, the typical timetable for promotion and tenure at academic institutions may not be amenable to the pursuit of CBPR as this methodology is more time-consuming than traditional research approaches. Researchers must contend with the time required to build sustainable partnerships and recruit and train community researchers, shifts in community priorities and leadership, and other unanticipated hurdles.^96,97^

In addition, although public health juggernauts such as the Institute of Medicine encourage academic researchers to foster community-academic partnerships that share the strengths of each and call for CBPR to be included among traditional curricula,^98,99^ most graduate programs do not require training in community-based research methodology. This may limit acceptability of CBPR as a viable, academically acceptable option. It certainly restricts the ability of untrained academic practitioners to effectively conduct CBPR research.^100^

## Conclusion

Implementing the CBPR framework with true fidelity is challenging. Across studies we found wide variation in the extent to which communities were involved in research activities, reflecting the diversity of CBPR partnerships, settings where SGM health research is conducted, and the inherent difficulty in adhering to the model. Although achieving a perfect score on the CBPR principles is difficult, if not impossible, the framework represents targets to strive for in the pursuit for more equitable and collaborative research conducted in SGM communities. Prioritizing this work can have a transformational impact on reducing the fundamental inequities that threaten SGM health.

## Supplementary Material

Supplemental data

Supplemental data

Supplemental data

## Supplementary Material

Supplementary Appendix SA1

Supplementary Appendix SA2

Supplementary Appendix SA3

## Abbreviations Used

AHRQ: Agency of Healthcare Research and Quality
AIDS: acquired immune deficiency syndrome
CBPR: community-based participatory research
GBMSM: gay, bisexual and other men who have sex with men
LGBTQ: lesbian, bisexual, transgender, queer
MSM: men who have sex with men
POC: people of color
RCTs: randomized controlled trials
SDOH: social determinants of health
SGM: sexual and gender minority
SGMY: sexual or gender minority youth
SOGIs: sexual orientation and gender identities
TGD: transgender and gender diverse
TGNC: transgender/gender non-conforming
TW: transgender women
