# Supplementary material for: A Systematic Literature Review of Community-Based Participatory Health Research with Sexual and Gender Minority Communities
Source: Health Equity. 2022 Aug 29;6(1):640–57. doi: 10.1089/heq.2022.0039 (PMC9448519; doi:10.1089/heq.2022.0039)
Supplement: Supplemental data [file Suppl_AppSA1.docx]

**Appendix Item 1. Search String**

(Community-based participatory research or CBPR or participatory research or action research or participatory action research or community driven research or action science)

AND

(LGBT or lesbian or gay or bisexual or transgender or queer or MSM or WSW or non-binary or gender fluid or gender queer or gender nonconforming)
