# Supplementary material for: A Systematic Literature Review of Community-Based Participatory Health Research with Sexual and Gender Minority Communities
Source: Health Equity. 2022 Aug 29;6(1):640–57. doi: 10.1089/heq.2022.0039 (PMC9448519; doi:10.1089/heq.2022.0039)
Supplement: Supplemental data [file Suppl_AppSA2.docx]

**Appendix Item 2. Data Extraction Tool**

**General information**

· Study ID

· Title (Title of paper / abstract / report that data are extracted from)

· Study funding sources

· Possible conflicts of interest for study authors

· Notes

**Characteristics of Included Studies**

· Methods

· Aim of study

· Study design

- - Randomized controlled trial
  - Non-randomized experimental study
  - Cohort study
  - Cross sectional study
  - Case control study
  - Qualitative research
  - Other

· Study setting

· Study start date

· Study end date

· Study intervention

· Duration of intervention

· Data collection methods

· Primary outcome(s)

· Secondary outcome(s)

· Methods used for analysis

· Key findings

· Participants

· Population description

· Inclusion criteria

· Exclusion criteria

· Method of recruitment of participants

o Phone

o Mail

o Clinic patients

o Convenience sampling

o Snowball sampling

o Random selection

o Other

· Total number of participants

· Response rate

· Retention rate

**Community Involvement**

· Duration of community involvement

· Nature of community involvement

· Evidence of shared decision-making between researchers and the community

· Evidence that study is designed to remove prior barriers to research participation

· Evidence that socioeconomic determinants of health are addressed by reducing barriers to research participation, as part of the intervention or as part of data collection

· Evidence that the research team was flexible to community needs and priorities during research implementation

· Evidence that the research effort contributed to individual or community capacity building

· Evidence of dissemination of findings to participants

· Evidence of application of findings to a health-related intervention or policy change

· Evidence of sustainability of research-related interventions in the community

**Did the community participate in....**

· ...selecting the research question?

- - Yes
  - No
  - Unknown

· ...developing the research proposal?

o Yes

o No

o Unknown

· ...assuming some financial responsibility for the project?

o Yes

o No

o Unknown

· ...designing the study?

o Yes

o No

o Unknown

· ...recruiting participants?

o Yes

o No

o Unknown

· ...retaining participants?

o Yes

o No

o Unknown

· ...developing surveys/instruments?

o Yes

o No

o Unknown

· ...data collection?

o Yes

o No

o Unknown

· ...developing intervention(s)

- - Yes
  - No
  - Unknown

· ...implementing intervention(s)?

o Yes

o No

o Unknown

· ...interpretation of findings?

o Yes

o No

o Unknown

· ...dissemination of findings?

o Yes

o No

o Unknown

· ...application of findings?

o Yes

o No

o Unknown

·
