# Supplementary material for: A Systematic Literature Review of Community-Based Participatory Health Research with Sexual and Gender Minority Communities
Source: Health Equity. 2022 Aug 29;6(1):640–57. doi: 10.1089/heq.2022.0039 (PMC9448519; doi:10.1089/heq.2022.0039)
Supplement: Supplemental data [file Suppl_AppSA3.docx]

**Appendix Item 3. Quality Assessment Tool**

· Research question clearly described

o Good (3)

o Fair (2)

o Poor/Insufficient information provided (1)

o NA

o *Extractors will also be able to add supporting text to justify their judgements*

· Study population adequately described

o Good (3)

o Fair (2)

o Poor/Insufficient information provided (1)

o NA

o *Extractors will also be able to add supporting text to justify their judgements*

· Study population appropriate to address stated research question

o Good (3)

o Fair (2)

o Poor/Insufficient information provided (1)

o NA

o *Extractors will also be able to add supporting text to justify their judgements*

· Study population representative of those to whom results might be generalized

o Good (3)

o Fair (2)

o Poor/Insufficient information provided (1)

o NA

o *Extractors will also be able to add supporting text to justify their judgements*

· Study population of adequate size to address research question

o Good (3)

o Fair (2)

o Poor/Insufficient information provided (1)

o NA

o *Extractors will also be able to add supporting text to justify their judgements*

· Evidence of structured guide/instrument to guide interviews/focus groups/observations (qualitative studies only)

o Good (3)

o Fair (2)

o Poor/Insufficient information provided (1)

o NA

o *Extractors will also be able to add supporting text to justify their judgements*

· Socio-cultural fit of interviewer/ leader/observer with participants (qualitative studies only)

o Good (3)

o Fair (2)

o Poor/Insufficient information provided (1)

o NA

o *Extractors will also be able to add supporting text to justify their judgements*

· Documentation of interviews/observations (qualitative studies only)

o Good (3)

o Fair (2)

o Poor/Insufficient information provided (1)

o NA

o *Extractors will also be able to add supporting text to justify their judgements*

· Systematic coding and analysis (qualitative studies only)

o Good (3)

o Fair (2)

o Poor/Insufficient information provided (1)

o NA

o *Extractors will also be able to add supporting text to justify their judgements*

· Randomized study?

o Yes

o No

o *Extractors will also be able to add supporting text to justify their judgements*

· Comparability of participants at baseline

o Good (3)

o Fair (2)

o Poor/Insufficient information provided (1)

o NA

o *Extractors will also be able to add supporting text to justify their judgements*

· Loss to follow-up

o Good (3)

o Fair (2)

o Poor/Insufficient information provided (1)

o NA

o *Extractors will also be able to add supporting text to justify their judgements*

· Intervention/exposure clearly described

o Good (3)

o Fair (2)

o Poor/Insufficient information provided (1)

o NA

o *Extractors will also be able to add supporting text to justify their judgements*

· Intervention/exposure dose assessed

o Good (3)

o Fair (2)

o Poor/Insufficient information provided (1)

o NA

o *Extractors will also be able to add supporting text to justify their judgements*

· Intervention feasible for implementation in larger population

o Good (3)

o Fair (2)

o Poor/Insufficient information provided (1)

o NA

o *Extractors will also be able to add supporting text to justify their judgements*

· Intervention delivered as planned (fidelity)

o Good (3)

o Fair (2)

o Poor/Insufficient information provided (1)

o NA

o *Extractors will also be able to add supporting text to justify their judgements*

· Clear distinction between comparison groups (avoidance of contamination, cross-over)

o Good (3)

o Fair (2)

o Poor/Insufficient information provided (1)

o NA

o *Extractors will also be able to add supporting text to justify their judgements*

· Primary outcome measures reflect research question

o Good (3)

o Fair (2)

o Poor/Insufficient information provided (1)

o NA

o *Extractors will also be able to add supporting text to justify their judgements*

· Primary outcome measures clearly defined

o Good (3)

o Fair (2)

o Poor/Insufficient information provided (1)

o NA

o *Extractors will also be able to add supporting text to justify their judgements*

· Primary outcome measures standardized

o Good (3)

o Fair (2)

o Poor/Insufficient information provided (1)

o NA

o *Extractors will also be able to add supporting text to justify their judgements*

· Primary outcome measures valid, reliable

o Good (3)

o Fair (2)

o Poor/Insufficient information provided (1)

o NA

o *Extractors will also be able to add supporting text to justify their judgements*

· Statistical analysis used intention to treat (clinical trials only)

o Good (3)

o Fair (2)

o Poor/Insufficient information provided (1)

o NA

o *Extractors will also be able to add supporting text to justify their judgements*

· Statistical analysis appropriate for study design (quantitative/mixed-method studies only)

o Good (3)

o Fair (2)

o Poor/Insufficient information provided (1)

o NA

o *Extractors will also be able to add supporting text to justify their judgements*

· Statistical analysis appropriately controlled for confounding (quantitative/mixed-method studies only)

o Good (3)

o Fair (2)

o Poor/Insufficient information provided (1)

o NA

o *Extractors will also be able to add supporting text to justify their judgements*

· Post intervention data collection (particularly interviews) blinded to study status

o Good (3)

o Fair (2)

o Poor/Insufficient information provided (1)

o NA

o *Extractors will also be able to add supporting text to justify their judgements*

· Statisticians blinded to study status (quantitative/mixed method studies only)

o Good (3)

o Fair (2)

o Poor/Insufficient information provided (1)

o NA

o *Extractors will also be able to add supporting text to justify their judgements*

· Possible bias due to funding source (highest potential for bias receives score of Poor (1))

o Good (3)

o Fair (2)

o Poor/Insufficient information provided (1)

o NA

o *Extractors will also be able to add supporting text to justify their judgements*

· Structure or mechanism for shared decision-making between researchers and the community

o Good (3)

o Fair (2)

o Poor/Insufficient information provided (1)

o NA

o *Extractors will also be able to add supporting text to justify their judgements*

· Study was designed to remove barriers to community participation in research

o Good (3)

o Fair (2)

o Poor/Insufficient information provided (1)

o NA

o *Extractors will also be able to add supporting text to justify their judgements*

· Socio-economic determinants of health were assessed through design of the study or intervention

o Good (3)

o Fair (2)

o Poor/Insufficient information provided (1)

o NA

o *Extractors will also be able to add supporting text to justify their judgements*

· Socio-economic determinants of health were addressed through design of the study or intervention

o Good (3)

o Fair (2)

o Poor/Insufficient information provided (1)

o NA

o *Extractors will also be able to add supporting text to justify their judgements*

· Research team was flexible to community needs and priorities during research implementation

o Good (3)

o Fair (2)

o Poor/Insufficient information provided (1)

o NA

o *Extractors will also be able to add supporting text to justify their judgements*

· Study's duration and purpose contributed to individual capacity building

o Good (3)

o Fair (2)

o Poor/Insufficient information provided (1)

o NA

o *Extractors will also be able to add supporting text to justify their judgements*

· Study's duration and purpose contributed to community capacity building

o Good (3)

o Fair (2)

o Poor/Insufficient information provided (1)

o NA

o *Extractors will also be able to add supporting text to justify their judgements*

· Findings were either used or intended to be used to address the original health concerns regarding dissemination to participants

o Good (3)

o Fair (2)

o Poor/Insufficient information provided (1)

o NA

o *Extractors will also be able to add supporting text to justify their judgements*

· Findings were either used or intended to be used to address the original health concerns regarding application to a health related intervention or policy change

o Good (3)

o Fair (2)

o Poor/Insufficient information provided (1)

o NA

o *Extractors will also be able to add supporting text to justify their judgements*

· Findings were either used or intended to be used to address the original health concerns regarding sustainability of research-related interventions in the community

o Good (3)

o Fair (2)

o Poor/Insufficient information provided (1)

o NA

o *Extractors will also be able to add supporting text to justify their judgements*
